# Sensory and motor correlates of frailty: dissociation between frailty phenotype and frailty index

**DOI:** 10.1186/s12877-022-03416-6

**Published:** 2022-09-15

**Authors:** Florian Beier, Martin Löffler, Frauke Nees, Lucrezia Hausner, Lutz Frölich, Herta Flor

**Affiliations:** 1 Institute of Cognitive and Clinical Neuroscience, Central Institute of Mental Health, Medical Faculty Mannheim, Heidelberg University, Square J5, 68159 Mannheim, Germany; 2 Institute of Medical Psychology and Medical Sociology, University Medical Center Schleswig-Holstein, Kiel University, Kiel, Germany; 3 Department of Geriatric Psychiatry, Central Institute of Mental Health, Medical Faculty Mannheim, Heidelberg University, Mannheim, Germany; 4 Department of Psychology, School of Social Sciences, University of Mannheim, Mannheim, Germany

**Keywords:** Frailty, Phenotype, Index, Sensory function, Motor function, Aging

## Abstract

**Background:**

Frailty has been associated with a decline in sensory and motor function. However, given that different frailty measures were shown to overlap but also differ in their diagnostic properties, sensory and motor correlates of frailty might be different depending on the operationalization of frailty. Our objective was to identify sensory and motor determinants of frailty and compare the results between frailty phenotype (FP) and frailty index (FI).

**Methods:**

Data from 44 pre-frail and frail subjects aged 65 and above were used. Frailty was measured using the FP and the FI. Sensory function in the visual, auditory, and tactile domain was assessed using visual acuity, absolute hearing threshold and mechanical detection threshold. Upper extremity motor performance was evaluated by the Purdue Pegboard Test and the Short Physical Performance Battery was used to assess lower extremity motor function. Multiple logistic regression models were employed to determine associations of sensory and motor function with frailty vs. pre-frailty for both frailty measures.

**Results:**

The frailty measures were moderately correlated (0.497, p ≤ 0.01) and had a Kappa agreement of 0.467 (*p* = 0.002). Using the FP, frailty was significantly associated with reduced upper extremity motor function only (OR = 0.50, 95% CI 0.29–0.87, *p* = 0.014). Frailty as assessed by the FI was significantly related to higher hearing thresholds (OR = 1.21, 95% CI 1.02–1.43, *p* = 0.027) and reduced lower extremity performance (OR = 0.32, 95% CI 0.13–0.77, *p* = 0.012).

**Conclusion:**

Frailty is related to reduced performance in measures of sensory and motor function. However, traditional measures of frailty might be differentially sensitive to capture sensory and motor decline, possibly contributing to the much-observed discordance between the diagnostic instruments. This should be taken into account by researchers and clinicians when planning and evaluating therapeutic interventions for frailty.

**Trial registration:**

ClinicalTrials.gov NCT03666039. Registered 11 September 2018 – Retrospectively registered.

## Background

Frailty describes a clinical condition that arises from a decline in multiple physiological systems and manifests in an increased vulnerability to minor stressor events, thereby increasing the risk for adverse health outcomes, including falls, hospitalization, and mortality [1]. The two most widely applied approaches to operationalize frailty are the frailty phenotype (FP) model and the cumulative deficit model [2, 3]. The phenotype model defines frailty on the basis of five physical criteria: unintentional weight loss, self-reported exhaustion, weak grip strength, slow gait speed and low physical activity level [2]. A person is classified as frail if three or more criteria are present, pre-frail if one or two criteria are present, or robust if none of the criteria is present. The cumulative deficit model in turn assumes that the more deficits a person has, the more likely that person is frail [3]. Here, frailty is expressed in terms of the frailty index (FI), which is determined by computing the ratio between the number of deficits present and the total number of deficits assessed. Both concepts were shown to be moderately correlated [4], and comparison studies revealed substantial diagnostic differences between the two scores [5]. For instance, studies using the FP reported lower prevalence of frailty than those using the FI [6]. Also, associations of frailty with age and mortality were stronger for the FP than the FI [7], while female gender, obesity and living alone were more strongly associated with the FI [8]. Apart from differences in diagnostic and predictive validity, the two frailty concepts also propose different processes in terms of the assumed underlying pathophysiological mechanisms of frailty. While the FP considers frailty as a biological and physical syndrome, the FI defines frailty as a multidimensional concept by emphasizing the quantity rather than the nature of health deficits [9]. Information on the characteristic correlates of these two frailty measures is therefore of both scientific and clinical value [5].

Among those critical mechanisms, the decline in motor abilities such as gait speed [10], postural control and balance [11, 12], as well as dexterity [13] was shown to greatly reduce mobility and limit daily activities [14], while elevating the risk for adverse events such as falls [15]. Apart from neuromuscular and musculoskeletal capacity, proper function of the motor system largely relies on the integration of multimodal sensory information. Deterioration of sensory systems is seen as a risk factor for age-related cognitive and motor decline and may precede the loss of mobility and independence by several years [14, 16]. Studies investigating sensory determinants of frailty found positive relationships between frailty and visual impairment [17, 18], hearing loss [19, 20] and impairments in tactile discrimination [21]. However, sensory impairment is considered in only a small number of frailty indices [22]. Adding the sensory domain to a frailty screening instrument has been demonstrated to change prevalence rates and to modify the risk profile associated with frailty [23]. These findings suggest that sensory and motor abilities might be differentially associated with frailty depending on the frailty measure used.

Therefore, in the current study, we measured sensory and motor abilities in a sample of (pre-)frail individuals to perform a physiological characterization of those deficits associated with frailty, as assessed by both the FP and FI. The objectives of the current study are twofold: first, we intended to examine the agreement between the FP and the FI in classifying individuals as frail; second, we aimed to determine sensory and motor correlates of frailty and compare these associations between the two frailty measures, given that the FP and FI represent different theoretical concepts of frailty.

## Methods

### Participants and procedure

Data are from a randomized controlled interventional study that has been described in detail elsewhere [24]. The aim of the interventional study was to compare a tablet-based sensorimotor training (experimental group) and a tablet-based relaxation training (control group) in subjects suffering from frailty. Ethics approval was obtained from the Ethics Committee of the Medical Faculty Mannheim, Heidelberg University. For the present cross-sectional analysis, only data from baseline assessments were used.

The present sample consists of N = 52 subjects who were recruited from collaborating geriatric centers, the general population via newspaper advertisements, info leaflets and online announcements and were pre-screened via telephone interviews for medical history, medication intake and activities of daily living to determine general eligibility. Subjects were included in the study if they (a) were aged 65 to 95 years and (b) fulfilled at least one of the five FP criteria, i.e. were classified as being pre-frail or frail, according to the FP model [2]. Subjects were excluded if they suffered from acute illnesses, severe neurological or mental disorders (i.e. depression), significant cognitive impairment (defined as a Mini Mental State Examination (MMSE) score of ≤ 24), or severe impairments in sensory abilities (i.e. visual acuity of < 0.1; mechanical detection threshold of > 512 mN; mean hearing threshold of > 60 dB; severe tinnitus symptomatology). See [24] for a detailed list of exclusion criteria.

### Frailty assessment

Frailty was assessed using the FP [2] and the FI [3]. The FP incorporates five different criteria: unintentional weight loss, exhaustion, low levels of physical activity, slow gait speed, and poor grip strength. Unintentional weight loss was evaluated based on self-reports asking the subject if they unintentionally lost 4.5 kg or more in weight within the past year. Exhaustion was assessed using two items from the German version of the Center for Epidemiologic Studies Depression Scale (CES-D) [25, 26]: “I could not get going”, and “I felt that everything I did was an effort”. Exhaustion was classified as present if a response of “occasionally” (3–4 days) or “most of the time” (5–7 days) regarding the past week was given to either question. Physical activity was measured asking subjects to state how much time they spent during the past two weeks doing 18 different leisure activities. The amount of time was converted into an estimate of the weekly energy expenditure in kilocalories and low physical activity was classified as present if the kilocalories expended per week fell below a cut-off value, stratified by gender (males: < 383 Kcals/week; females: < 270 Kcals/week). Gait speed was determined by measuring the time taken to walk 4.57 m at usual pace, using walking aids if needed. Cut-off points were stratified by gender and height (males: height ≤ 173 cm: ≥ 7 s, height > 173 cm: ≥ 6 s; females: height ≤ 159 cm: ≥ 7 s, height > 159 cm: ≥ 6 s). Grip strength was measured in kg using a Jamar hand dynamometer (Patterson Medical, Cedarburg, WI, USA). Maximal grip strength at the dominant hand was averaged across three trials and cut-off scores were stratified by gender and body mass index (males: BMI ≤ 24: ≤ 29 kg, BMI 24.1 – 26: ≤ 30 kg, BMI 26.1 – 28: ≤ 30 kg, BMI > 28: ≤ 32 kg; females: BMI ≤ 23: ≤ 17 kg, BMI 23.1 – 26: ≤ 17.3 kg, BMI 26.1 – 29: ≤ 18 kg, BMI > 29: ≤ 21 kg). Subjects fulfilling three or more FP criteria were classified as frail while subjects fulfilling one or two criteria were classified as pre-frail.

To determine the FI, we used 40 deficit variables and cut-off points as developed by Searle et al. [3] consisting of physical, psychological, social and cognitive items and reported comorbidity excluding shoulder strength measurement due to feasibility reasons. Each deficit was dichotomized with a score of 0 representing absence of the deficit and 1 representing full presence of the deficit. For some items, intermediate scores were used to allow for a finer grading of the respective deficit. The FI was calculated by summing all deficits and dividing by the total number of deficits, resulting in a total score ranging from 0 (no deficit present) to 1 (all deficits present). Using previously reported cut-off points [4, 27], individuals with a FI score > 0.25 were considered as frail and those with a score ≤ 0.25 were considered as pre-frail.

### Sensory assessment

#### Visual acuity

Binocular visual acuity was assessed using the automated Freiburg Visual Acuity and Contrast Test (FrACT) [28, 29]. The test was performed in an artificially lit room and test stimuli were presented on a 15-inch LCD monitor (resolution 1280 × 800) at a distance of 150 cm. All subjects were tested without wearing any visual aids while subjects who used visual aids to correct their vision were additionally tested while wearing their visual aids. For those subjects, the better one of the two scores (with or without visual aids) was considered for the subsequent analyses. In the visual acuity test, 18 black Landolt-C optotypes were successively presented one at a time randomly at one of eight possible orientations against a white background and subjects were to indicate the orientation of the optotype in a forced-choice manner. The size of the optotype was adapted in each trial according to a staircase best-PEST procedure [30, 31]. Visual acuity was quantified using the logMAR score which is defined as the negative logarithm of the decimal visual acuity score (logMAR = -log(VA)). Thus, lower logMAR scores represent higher visual acuity.

#### Auditory perception thresholds

To assess auditory perception thresholds, pure-tone audiometry was performed in a sound-shielded and anechoic booth using a screening audiometer (MA 25, MAICO Diagnostics GmbH, Berlin, Germany). Subjects were tested without wearing any hearing aids and single tones were presented via headphones separately to the right and left ear in a counterbalanced manner. The tones were manually given by the experimenter and subjects were asked to press a button whenever they perceived a tone. For each frequency of 500, 1000, 2000 and 4000 Hz, absolute hearing thresholds in decibel (dB) were determined using a staircase procedure [32]. Absolute hearing thresholds were then averaged across the four frequencies separately for the right and left ear. For the subsequent analyses, the hearing threshold of the better hearing ear was considered.

#### Somatosensory perception thresholds

Touch thresholds were determined by stimulating the fingertip of the index finger of the dominant hand using von Frey filaments (Marstocknervtest, Marburg, Germany). Possible touch forces ranged from 0.25 to 512 mN in a logarithmic scale. During the test, subjects were asked to close their eyes and to verbally indicate whenever they perceived a sensation on their skin. The filaments were manually applied by the experimenter perpendicular to the subject’s skin. A staircase procedure [33, 34] was applied resulting in five values for upper and lower boundaries, respectively, that were averaged to obtain the touch threshold. Here, lower scores reflect enhanced sensation.

### Motor assessment

#### Upper extremity function

To assess upper extremity function, we used the Purdue Pegboard Test (PPT) [35] which measures fine and gross motor dexterity and coordination of hands, fingers, and arms [36]. Subjects had to use their dominant hand to place small metal pegs into holes one at a time from top to bottom as fast as possible within a 30-s epoch. Three runs were administered and the number of correctly placed metal pegs was averaged across runs.

### Lower extremity function

Lower extremity function was assessed using the Short Physical Performance Battery (SPPB) [37] comprising timed measures of balance, walking speed, and sit-to-stand ability. For the balance test, subjects had to maintain their feet in side-by-side, semitandem and tandem position for 10 s each. For the walking speed test, subjects were asked to walk at their usual speed over a 4-m distance, using walking aids if needed. For the sit-to-stand test, subjects were asked to stand up from a chair and sit down five times in a row as quickly as possible without using their arms while the time to perform the task was recorded. Performance measures of the individual subtests were then converted into a score ranging from 0 to 4 and were summed up to compute the total SPPB score ranging from 0 to 12.

### Additional measures and covariates

Sociodemographic data of each subject were collected through a verbally administered questionnaire requesting information about age, gender, somatic co-morbidities, handedness and use of sensory aids. Height, weight and body mass index were obtained through a physical examination performed by a study physician. The MMSE was used to screen for cognitive impairment. Depressive symptoms were assessed using the German version of the 20-item Center for Epidemiologic Studies Depression Scale (CES-D) [25, 26].

### Statistical analyses

Analyses were carried out using SPSS version 25 (Armonk, NY: IBM Corp.). Continuous variables were checked for normal distribution using Kolmogorov–Smirnov tests. Descriptive characteristics of each variable are reported as mean and standard deviation (SD) for continuous variables and frequency and percentages for categorical variables. Descriptive variables were additionally stratified by gender and gender differences were examined using Student’s t-tests for normally distributed variables, Mann–Whitney-U-tests for non-normally distributed variables and Pearson-chi²-tests for categorical variables. Additionally, descriptive variables were contrasted between pre-frail and frail for both frailty measures. Agreement between the two frailty measures was assessed using a kappa statistic and frailty prevalence was compared using the McNemar test. To examine associations of the demographic, sensory and motor variables with frailty as well as between the two frailty measures, correlation coefficients were calculated using Pearson correlations for normally distributed variables and Spearman correlations for non-normally distributed variables. To identify independent determinants of frailty, hierarchical multiple logistic regression models were calculated for each of the two frailty measures, using frailty status (pre-frail, frail) as outcome. In the first block, age and gender were entered into the analyses as covariates to account for the known relationship between age, gender and frailty. In addition, depressive symptoms (CES-D score) were added, which have previously been associated with both sensory impairment and frailty [38–41]. In the second block, sensory and motor variables were added to the analyses to determine the respective associations with frailty. For the regression models, odds ratio (OR), 95% confidence intervals (CI) and Nagelkerke’s R^2^ along with the *p*-value for the model are reported. A significance level of 0.05 was used for all analyses.

## Results

### Sample characteristics

Of the total number of 52 subjects, 8 subjects were excluded from the analyses because they had incomplete data sets due to study withdrawal. The characteristics of the remaining 44 subjects are presented in Table 1. The mean age was 80.4 (SD: 5.5) years, ranging from 68.6 to 91.9 years, and 72.7% (*n* = 32) were women. Regarding frailty assessment, the mean number of positive FP criteria, averaged across all subjects, was 2.3 (SD: 0.8). Eight (18.2%), 20 (45.5%), 13 (29.5%) and 3 (6.8%) subjects had 1, 2, 3, or 4 positive FP criteria, respectively, while none fulfilled all 5 of the criteria. Thus, 28 (63.6%) subjects were classified as pre-frail and 16 (36.4%) were considered to be frail. The FI had a mean value of 0.23 (SD: 0.09), and ranged from 0.08 to 0.47. Using a cut point of 0.25, 27 (61.4%) subjects were classified as pre-frail and 17 (38.6%) were considered to be frail. With respect to gender differences, the mean FI score was significantly lower for males than females (*p* = 0.005), suggesting that males were classified as less frail, while there was no gender difference in the number of positive FP criteria (*p* = 0.630). The mean value of body mass index, MMSE, and CES-D was 28.5 (SD: 6.4), 28.7 (SD: 1.5), and 16.0 (SD: 8.3), respectively. For the sensory assessment, the mean value of visual acuity (logMAR), hearing threshold (dB), and mechanical detection threshold (mN) was 0.19 (SD: 0.17), 33.3 (SD: 12.0), and 0.74 (SD: 1.04), respectively. Regarding motor function, mean PPT performance was 9.8 (SD: 2.3) and mean SPPB score was 7.4 (SD: 2.3). Males had a significantly lower body mass index (*p* = 0.015) and higher scores of lower extremity function (SPPB; *p* = 0.003) compared to females. When comparing those classified as frail vs. pre-frail using the FP and FI, frail subjects had higher scores of depression and reduced scores of lower extremity function compared to pre-frail subjects (see Table 2). Additionally, frail subjects demonstrated reduced upper motor function (PPT) according to the FP and were older when categorized with the FI.

**Table 1:** Characteristics of the study sample, stratified by gender

	All (*n* = 44)	Female (*n* = 32)	Male (*n* = 12)	*p*-value
Age, years, mean ± SD	80.4 ± 5.5	80.8 ± 5.4	79.4 ± 6.0	0.462
Body mass index, kg/m^2^, mean ± SD	28.5 ± 6.4	30.0 ± 6.7	24.7 ± 3.8	0.015
MMSE, mean ± SD	28.7 ± 1.5	28.9 ± 1.4	28.3 ± 1.7	0.267
CES-D, mean ± SD	16.0 ± 8.3	17.4 ± 8.5	12.4 ± 6.6	0.077
Visual acuity, logMAR, mean ± SD	0.19 ± 0.17	0.22 ± 0.18	0.11 ± 0.13	0.059
Hearing threshold, dB, mean ± SD	33.3 ± 12.0	32.9 ± 11.7	34.5 ± 13.2	0.805
Mechanical detection threshold, mN, mean ± SD	0.74 ± 1.04	0.67 ± 0.80	0.91 ± 1.53	0.490
PPT score, mean ± SD	9.8 ± 2.3	9.8 ± 2.5	9.7 ± 1.8	0.924
SPPB score, mean ± SD	7.4 ± 2.3	6.8 ± 2.2	9.1 ± 1.9	0.003
FP criteria, mean ± SD	2.3 ± 0.8	2.3 ± 0.9	2.2 ± 0.8	0.630
*FP criteria (number), n (%)*				
1	8 (18.2)	6 (18.8)	2 (16.7)	-
2	20 (45.5)	13 (40.6)	7 (58.3)	-
3	13 (29.5)	11 (34.4)	2 (16.7)	-
4	3 (6.8)	2 (6.3)	1 (8.3)	-
5	0 (0.0)	0 (0.0)	0 (0.0)	-
*FP criteria (type), n (%)*				
Weight loss	11 (25.0)	6 (18.8)	5 (41.7)	0.118
Exhaustion	23 (52.3)	16 (50.0)	7 (58.3)	0.622
Physical activity	6 (13.6)	5 (15.6)	1 (8.3)	0.530
Slow gait speed	28 (63.6)	21 (65.6)	7 (58.3)	0.654
Low grip strength	31 (70.5)	25 (78.1)	6 (50.0)	0.069
*Categories according to the FP, n (%)*				
Pre-frail	28 (63.6)	19 (59.4)	9 (75.0)	0.337
Frail	16 (36.4)	13 (40.6)	3 (25.0)	
FI, mean ± SD	0.23 ± 0.09	0.25 ± 0.08	0.17 ± 0.06	0.005
*Categories according to the FI, n (%)*				
Pre-frail	27 (61.4)	16 (50.0)	11 (91.7)	0.011
Frail	17 (38.6)	16 (50.0)	1 (8.3)	

Data are shown as mean ± standard deviation or n (%). *CES-D* Center for Epidemiologic Studies Depression Scale, *FI* Frailty index, *FP* Frailty phenotype, *MMSE* Mini-mental state examination, *PPT* Purdue Pegboard Test, *SD* Standard deviation, *SPPB* Short physical performance battery

**Table 2:** Characteristics of the study sample, stratified by frailty status for both frailty measures

	FP			FI		
	Pre-frail (*n* = 28)	Frail (*n* = 16)	*p*-value	Pre-frail (*n* = 27)	Frail (*n* = 17)	*p*-value
Age, years, mean ± SD	79.9 ± 5.6	81.3 ± 5.5	0.412	78.9 ± 5.5	82.8 ± 4.8	0.021
Body mass index, kg/m^2^, mean ± SD	29.1 ± 6.2	27.6 ± 6.8	0.469	27.9 ± 6.5	29.6 ± 6.4	0.403
MMSE, mean ± SD	28.9 ± 1.5	28.5 ± 1.4	0.238	28.8 ± 1.6	28.7 ± 1.3	0.362
CES-D, mean ± SD	13.3 ± 6.7	20.8 ± 8.9	0.003	13.3 ± 6.3	20.3 ± 9.4	0.012
Visual acuity, logMAR, mean ± SD	0.20 ± 0.17	0.18 ± 0.18	0.816	0.16 ± 0.13	0.24 ± 0.22	0.161
Hearing threshold, dB, mean ± SD	34.0 ± 12.4	32.0 ± 11.4	0.855	30.7 ± 11.2	37.4 ± 12.4	0.058
Mechanical detection threshold, mN, mean ± SD	0.69 ± 1.12	0.82 ± 0.91	0.486	0.79 ± 1.21	0.66 ± 0.71	0.621
PPT score, mean ± SD	10.6 ± 1.9	8.3 ± 2.3	0.001	10.2 ± 2.2	9.0 ± 2.5	0.075
SPPB score, mean ± SD	8.1 ± 1.7	6.2 ± 2.7	0.017	8.4 ± 1.7	5.8 ± 2.3	< 0.001

Data are shown as mean ± standard deviation. *CES-D* Center for Epidemiologic Studies Depression Scale, *FI* Frailty index, *FP* Frailty phenotype, *MMSE* Mini-mental state examination, *PPT* Purdue Pegboard Test, *SD* Standard deviation, *SPPB* Short physical performance battery

### Relationship between frailty measures and demographic, sensory and motor variables

**Fig. 1:**
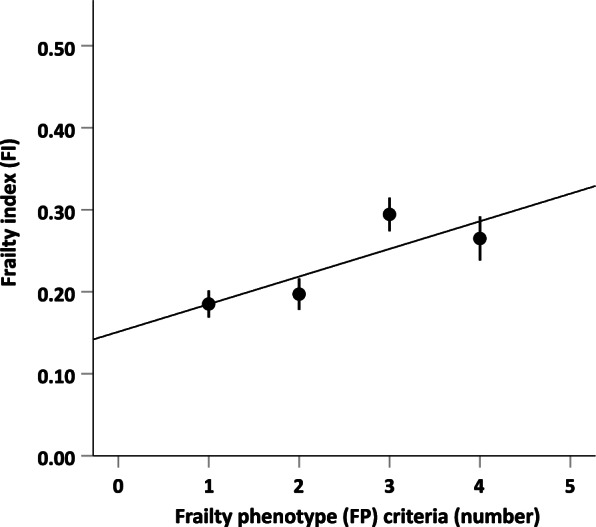
Relationship between FP (number of deficits) and FI (mean score). Error bars represent standard errors of the mean FI. Note that subjects had at least one positive FP criterion due to study inclusion criteria. None of the subjects had five FP criteria

Observed agreement between the two frailty measures in classifying individuals as pre-frail or frail was 75.0% (see Table 3) with a Kappa statistic of 0.467 (*p* = 0.002). Frailty prevalence (FP: 36.4%; FI: 38.6%) did not significantly differ between the two measures (*p* = 1.00). Table 4 displays the correlations among the two frailty measures and demographic, sensory and motor variables. There was a moderate positive correlation between the mean FI score and the number of positive FP criteria (0.497, *p* = 0.001; see Fig. 1). Frailty as determined by both the FP criteria and the FI was significantly negatively associated with upper (PPT; FP: -0.417, p = 0.005; FI: -0.430, *p* = 0.004) and lower extremity function (SPPB; FP: -0.392, *p* = 0.009; FI: -0.645, *p* < 0.001). Unlike the FP, the FI significantly positively correlated with depression (0.532, *p* < 0.001). None of the two frailty measures was significantly associated with measures of visual, auditory, or somatosensory abilities (all p ≥ 0.155). For the demographic, sensory and motor variables, there were significant associations between visual acuity and age (0.434, *p* = 0.003), visual acuity and hearing threshold (0.313, *p* = 0.039), hearing threshold and age (0.452, *p* = 0.002), mechanical detection threshold and upper motor function (-0.323, *p* = 0.032) and between upper and lower motor function (0.329, *p* = 0.029).

**Table 3:** Proportion of participants within the FP and FI categories, n (%)

		FI		Total
		Pre-frail	Frail	
FP	Pre-frail	22 (50.0)	6 (13.6)	28 (63.6)
	Frail	5 (11.4)	11 (25.0)	16 (36.4)
		27 (61.4)	17 (38.6)	44 (100)

*FI* Frailty index, *FP* Frailty phenotype

**Table 4:** Correlations between frailty and demographic, sensory and motor variables

	FP criteria (number)	FI	Age (years)	Body mass index	MMSE	CES-D	Visual acuity	Hearing threshold	Mechan-ical detection threshold	Purdue Pegboard score	SPPB score
FP criteria (number)	1.000										
FI	0.497**	1.000									
Age (years)	0.242	0.269	1.000								
Body mass index	-0.104	0.250	-0.120	1.000							
MMSE	-0.147	-0.032	-0.377*	0.232	1.000						
CES-D	0.293	0.532**	0.149	0.077	-0.122	1.000					
Visual acuity	-0.017	0.218	0.434**	0.205	-0.220	-0.007	1.000				
Hearing threshold	0.000	0.130	0.452**	0.030	-0.140	0.011	0.313*	1.000			
Mechanical detection threshold	0.168	-0.030	0.144	-0.103	-0.278	0.087	-0.078	0.288	1.000		
PPT score	-0.417**	-0.430**	-0.282	-0.021	0.534**	-0.225	-0.143	-0.095	-0.323*	1.000	
SPPB score	-0.392**	-0.645**	-0.229	-0.094	-0.012	-0.356*	-0.196	0.002	-0.142	0.329*	1.000

*CES-D* Center for Epidemiologic Studies Depression Scale, *FI* Frailty index, *FP* Frailty phenotype, *MMSE* Mini-mental state examination, *PPT* Purdue Pegboard Test, *SPPB* Short physical performance battery

^*^p ≤ 0.05; ** *p* ≤ 0.01

### Hierarchical multiple logistic regression models

The results of the hierarchical multiple logistic regression analyses of covariates and sensory and motor variables on frailty are depicted in Table 5.

**Table 5:** Results of the hierarchical multiple logistic regression analyses for relationships of demographic, sensory and motor variables with FP and FI

Independent variables	FP		FI	
	OR (95% CI)	p-value	OR (95% CI)	*p*-value
**Block 1**				
Age	1.03 (0.90–1.17)	0.666	1.18 (1.01–1.38)	0.042
Gender	1.19 (0.23–6.10)	0.838	9.76 (0.94–101.33)	0.056
CES-D	1.13 (1.03–1.24)	0.013	1.11 (1.00–1.23)	0.044
	R^2^ = 0.254	0.029	R^2^ = 0.447	0.001
**Block 2**				
Age	0.94 (0.77–1.16)	0.583	1.07 (0.81–1.40)	0.649
Gender	1.36 (0.15–12.28)	0.785	56.89 (0.88–3660.12)	0.057
CES-D	1.11 (0.99–1.25)	0.087	1.20 (1.01–1.44)	0.040
Visual acuity	0.22 (0.00–84.65)	0.614	0.01 (0.00–275.16)	0.397
Hearing threshold	1.00 (0.91–1.09)	0.928	1.21 (1.02–1.43)	0.027
Mechanical detection threshold	0.96 (0.28–3.28)	0.951	0.11 (0.01–1.49)	0.096
PPT score	0.50 (0.29–0.87)	0.014	0.64 (0.32–1.31)	0.223
SPPB score	0.69 (0.44–1.07)	0.100	0.32 (0.13–0.77)	0.012
	R^2^ = 0.542	0.005	R^2^ = 0.748	< 0.001
	R^2^ change	0.022	R^2^ change	0.003

*CES-D* Center for Epidemiologic Studies Depression Scale, *FI* Frailty index, *FP* Frailty phenotype, *PPT* Purdue Pegboard Test, *SPPB* Short physical performance battery

#### Frailty phenotype

In the first block, only depression (OR = 1.13, 95% CI 1.03–1.24, *p* = 0.013) was significantly associated with pre-frail vs. frail as classified by the FP, with the covariate model explaining 25.4% of the variance (*p* = 0.029). In the second block, upper extremity function as assessed by the PPT score (OR = 0.50, 95% CI 0.29–0.87, *p* = 0.014) was independently associated with frailty and the total amount of variance explained by the model was significantly increased to 54.2% (*p* = 0.005).

#### Frailty index

Regarding the FI, the covariate model explained 44.7% of variance (*p* = 0.001) and age (OR = 1.18, 95% CI 1.01–1.38, *p* = 0.042) and depression (OR = 1.11, 95% CI 1.00–1.23, *p* = 0.044) were independently associated with frailty. In the second block, frailty was significantly associated with depression (OR = 1.20, 95% CI 1.01–1.44, *p* = 0.040), hearing threshold (OR = 1.21, 95% CI 1.02–1.43, *p* = 0.027) and lower extremity function as determined by the SPPB score (OR = 0.32, 95% CI 0.13–0.77, *p* = 0.012). Adding the sensory and motor variables significantly improved the predictive value of the model (*p* = 0.003) compared to the covariate model and raised the amount of explained variance to 74.8% (*p* < 0.001).

## Discussion

The objectives of the present study were to examine the agreement between the FP and the FI in classifying individuals as frail and to identify sensory and motor correlates of frailty and compare these associations between the two frailty measures. Our findings demonstrate that the FP and the FI moderately agree in classifying the same individuals as either pre-frail or frail, but that there is heterogeneity when determining sensory and motor correlates of frailty. Given that frailty is a potentially reversible state [42], the identification of characteristic correlates and knowledge about the underlying deficits is necessary for offering timely and appropriate interventions.

### Diagnostic agreement between FP and FI

In our study, we found a moderate Kappa agreement of 0.467 between the two frailty measures, which is consistent with previous literature reporting agreement ranging from 0.428 [43] to 0.51 [9] when dichotomized frailty measures and a cut point of 0.25 for the FI are used. We also observed a significant moderate correlation in continuous scores of 0.497 in accordance with earlier studies reporting correlations ranging from 0.361 [44] to 0.76 [8]. In our cross-sectional analysis, agreement between measures was potentially strengthened by the fact that subjects had to fulfill at least one FP criterion to be included in the primary training study while previous cross-sectional studies also considered robust non-frail individuals [8, 9, 43, 45]. There is evidence that the FI compared to the FP discriminates better at the lower end of the frailty continuum [9, 45, 46] and classifies a larger number of individuals as frail, resulting in higher prevalence rates, [8, 44, 45, 47]. Therefore, reducing variability at the lower end of the frailty continuum by including subjects that are at least pre-frail might also be a possible explanation why frailty prevalence did not substantially differ between the two measures in the current study.

Our results with the FI correspond to studies which demonstrated that at the same age, women have significantly greater frailty than men [6, 48–51]. Various explanations for increased frailty in females have been discussed and tested in the literature, including biological, behavioral and social factors [47, 48, 52, 53], indicating that females compared to males are likely to acquire more health deficits overall and have these deficits for a longer period of time [49]. While greater frailty in women compared to men has been consistently demonstrated across various frailty measures [48, 49, 54], we observed gender differences in frailty only for the FI, but not for the FP. There might be several possible explanations for this finding. First, it might be that the higher FI in women compared to men represents an increased vulnerability of women, which however is not captured by the FP. In line with that, previous studies found associations between the FI and adverse health measures even in subjects who were classified as non-frail by the FP [9]. Thus, one might argue that the higher FI scores in women result from the fact that, due to its continuous nature, the FI captures an increased subclinical vulnerability before the full picture of frailty becomes manifested in the FP [45, 55]. Second, it might be that gender-specific differences in the underlying pathological mechanisms of frailty are captured in different ways by the two frailty measures. More specifically, the FP considers frailty as a biological and physical syndrome whereas the FI defines frailty by the quantity rather than the nature of health deficits [9]. Cross-sectional analyses suggested that the underlying determinants of frailty are more complex and interrelated in women compared to men [54, 56]. Given that the FI includes mental health, medical conditions as well as indicators of disability while the FP does not, it might be that the FI, compared to the FP, superiorly detects the multidimensional risk and vulnerability in women that underlies the physical expression of frailty [7, 9, 57].

Together, our results support the perspective that both frailty instruments share some common characteristics but also slightly differ from each other in detecting frailty in the same population [8, 9, 44], raising the question as to the characteristic correlates of the two frailty concepts.

### Sensory and motor correlates of frailty depending on frailty measure

Our multiple regression analyses revealed overlapping as well as non-overlapping associations of demographic, sensory and motor variables with frailty vs. pre-frailty as a function of the frailty measure. We observed strong associations of reduced physical and motor performance with greater frailty for both frailty approaches. The total regression model for the FP demonstrated a significant relationship between frailty and dexterity performance whereas the FI was associated with lower extremity performance. Previous evidence demonstrated that upper limb dexterity performance might differentiate robust from pre-frail/frail individuals [58]. In our descriptive analyses, we also found a significantly lower dexterity performance in frail vs. pre-frail subjects as determined by the FP suggesting that also within the group of pre-frail and frail subjects, upper extremity function is related to the degree of frailty. These results fit well with earlier research showing that reduced upper extremity control might be a marker of an increased risk for frailty and dependency [59, 60]. Likewise, the observed relationship between SPPB performance and frailty as determined by the FI is consistent with prior evidence stating that impaired lower extremity performance is a key indicator of frailty and SPPB performance has previously been shown to reliably identify frail individuals [57, 61, 62].

However, while both PPT and SPPB performance demonstrated strong linear associations with both frailty measures in the bivariate correlation analyses, only PPT performance was found to be an independent predictor of the FP, and only SPPB performance was independently associated with the FI in the multiple regression models. This finding seems to indicate that there are common factors and mechanisms shared among upper and lower extremity motor performance as well as with the other domains inspected in the models (e.g. sensory abilities, depression, gender) that are associated with frailty. Thus, there is more to motor decline in frailty than motor variables alone and further factors should be taken into account to map the complex and interacting mechanisms that underlie physical and motor decline in frailty [13].

We found a significant independent association between hearing threshold and the FI. Previous cross-sectional studies found that perceived hearing impairment was independently associated with frailty in older women [19] and helped to predict frailty risk in community-living older persons [63]. Similarly, hearing impairment has been independently associated with frailty-related deficits, including gait speed [64], increased falls [65, 66], depression [67], hospitalizations and mortality [68]. The mechanisms that could underlie an association between hearing impairment and frailty are still not fully understood. Degradation of shared neurophysiological pathways, including neurodegeneration, microvascular disease and systemic inflammation might contribute to both hearing disability and frailty [66, 69–71]. Alternatively, hearing impairment might potentially affect frailty through mediating effects of cognitive impairment [72, 73], social isolation [74], and depression [75, 76]. Notably, we only found an association between hearing ability and frailty with the FI, but not the FP. This is not necessarily in contrast to prior findings, given the methodological heterogeneity in previous studies. While some studies assessed hearing impairment through self-report [19, 63], we obtained behavioral fine-grained sensory measures by using pure-tone audiometry. This reduced the impact of potential self-report bias which might depend on the individuals’ insight regarding chronic disease [77] and on the tendency to generalize the rating from a diminished function in one sense to the other senses as well [78]. Moreover, some studies used non-traditional definitions of frailty [66] whereas we used two well established frailty measures that are also most widely used in geriatric practice. Our observation suggests that the FI might be more sensitive than the FP in capturing sensory decline that is associated with frailty. For instance, the FI contains items that might reflect direct or indirect effects of hearing disability on frailty, such as everyday function, mood, cognitive abilities and previous diseases. In this regard, the observed relationship might also be a function of increased comorbidities and accumulation of multidimensional deficits as assessed by the FI. However, despite the fact that age-related sensory impairments are strongly associated with physical decline [79, 80], the lack of associations between sensory performance and the FP in our analyses suggests that the precise mechanisms underlying the sensory impairment – physical frailty relationship may be more complex than those reflected by the physical FP. Assuming that traditional frailty measures might be more or less sensitive in capturing the contribution of sensory impairment to frailty, our results support previous proposals that the evaluation of sensory abilities should be included in frailty assessment protocols [21, 23, 81].

Depression as assessed by the CES-D was identified as a significant covariate for FI. There is ample evidence about the association of depression with frailty in older age suggesting that associations between depression and frailty might be driven by various common characteristics, such as exhaustion, slowness and weight loss [38, 39]. For instance, we found a significant negative correlation between depression and SPPB scores which is consistent with earlier findings reporting a relationship between depression and lower-extremity performance and mobility [82]. However, it has been argued that physical symptoms and functional impairment in elderly may inflate scores on depression measures including somatic items, such as the CES-D, compared to other measures such as the Geriatric Depression Scale (GDS), which contains no somatic items [83]. When replacing the CES-D score with the GDS score in our analyses, depression is no longer found to be independently related to frailty status for either frailty measure, but the pattern of independent relationships between sensory and motor determinants and frailty as well as the differences observed between the two frailty indicators does not substantially change (data not shown). This implies that the relationship between depression and frailty in our analyses might be driven by the overlap of somatic symptoms in the measures, which however did not bias our main findings. Importantly, associations of depression with frailty were also found when the measures used to determine one syndrome were adjusted for the characteristics of the other syndrome [38], suggesting that it is not the shared characteristics alone that explain the increased severity of depression in frail compared to non-frail depressed older individuals [38]. Previous reviews suggested a bidirectional relationship between depression and frailty [39, 84] and evidence pointed to common pathophysiological mechanisms, such as low-grade inflammation [85, 86], that might underlie the relationship between depression and frailty. The fact that we found a significant independent association only between depression and frailty as assessed with the FI but not the FP might be surprising given that higher scores of depression were found in frail vs. pre-frail individuals as determined by either frailty measure. However, it should be noted that, given that the independent association between depression and the FP in the multivariate analysis almost reached significance (*p* = 0.087), we cannot exclude the possibility that we failed to find an existing effect due to insufficient statistical power.

### Strengths and limitations

Our study has several strengths. First, we used validated behavioral measures to assess sensory, motor and physical performance, thereby reducing the impact of potential self-report bias. Second, we demonstrated that conclusions about the underlying impairments of a person classified as frail should be made with caution because the identification of frailty and potentially modifiable determinants, particularly physical performance, sensory impairment and depression, is influenced by the frailty measure and construct employed. Our findings provide further evidence that frailty measures should not be used interchangeably [5] and that multiple dimensions should be taken into account when diagnosing and treating the frailty syndrome.

Our study is also subject to limitations. Due to the cross-sectional nature of the analyses, we cannot determine the temporal relationship and causal or mechanistic pathways underlying the relationship of sensory and motor abilities with frailty. Taking into account the small number of subjects in the sample, our results should therefore be considered as preliminary. Moreover, the fact that our analyses were performed on baseline data from individuals participating in a frailty intervention study might have promoted selection and exclusion bias in our sample. For instance, we selected subjects without significant cognitive impairment (MMSE > 24) while frailty and cognitive dysfunction were demonstrated to be significantly associated in the population [87, 88]. Also, the fact that subjects had to fulfil at least one positive FP criterion excluded robust (i.e. non-frail) individuals from the sample while the need for willingness and ability to participate in a multi-month intervention program might have favored the inclusion of less frail individuals [49]. Thus, the relationships that we found in a sample presumably located in the lower to middle range of the frailty continuum still need to be examined in robust elderly individuals and individuals located at the upper end of the frailty continuum. Therefore, generalizability of our results may be limited, and it will still have to be examined whether our results apply to other frailty measures, to the use of different cutoffs for frailty and pre-frailty, to the use of different measures of sensory and motor performance or to different subject populations.

## Conclusion

Based on our results, we assume that the differences by which the FP and FI capture frailty-related sensory, motor and psychological impairment could provide an explanation for the frequently observed discordance in identifying frailty. These differences in diagnostic properties have implications for researchers and clinicians, since the choice of the instrument may influence the accurate identification of frailty and the planning of interventions in individuals suffering from different impairments. Therefore, the objectives of the use should be taken into account in order to select the appropriate instrument. Our cross-sectional analyses indicate that frailty is a multidimensional and complex syndrome and future representative studies involving large-scale longitudinal data of objective sensory and motor performance in community-dwelling as well as institutionalized robust, pre-frail and frail individuals will be needed to identify the temporal and causal mechanisms underlying the relationship of sensory and motor impairment with frailty. The understanding of mechanistic pathways is imperative for considering the heterogeneity of changes in function, for refining existing diagnostic systems and measures of frailty and for developing individualized treatment plans. In this context, sensory and motor determinants of frailty, which are potentially modifiable, might represent useful targets for the development of effective prevention and treatment strategies to maintain function and independence in old age.

## Data Availability

The dataset used and analyzed in the current study will be available from the corresponding author on reasonable request.

## Abbreviations

CES-D: Center for Epidemiologic Studies Depression Scale
CI: Confidence interval
dB: Decibel
FI: Frailty index
FP: Frailty phenotype
kg: Kilogram
MMSE: Mini Mental State Examination
mN: Millinewton
OR: Odds Ratio
PPT: Purdue Pegboard Test
SD: Standard deviation
SPPB: Short Physical Performance Battery
